# Molecular Epidemiology of Group A Streptococcus Infections in Cambodian Children, 2007–2012

**DOI:** 10.1097/INF.0000000000000878

**Published:** 2015-11-12

**Authors:** Paul Turner, Pises Ngeth, Claudia Turner, Sena Sao, Nicholas P. J. Day, Ciara Baker, Andrew C. Steer, Pierre R. Smeesters

**Affiliations:** Cambodia-Oxford Medical Research Unit Angkor Hospital for Children, Siem Reap, Cambodia, Centre for Tropical Medicine and Global Health Nuffield Department of Medicine University of Oxford Oxford, United Kingdom; Angkor Hospital for Children Siem Reap, Cambodia; Cambodia-Oxford Medical Research Unit Angkor Hospital for Children, Siem Reap, Cambodia Centre for Tropical Medicine and Global Health Nuffield Department of Medicine University of Oxford Oxford, United Kingdom; Angkor Hospital for Children Siem Reap, Cambodia; Centre for Tropical Medicine and Global Health Nuffield Department of Medicine University of Oxford Oxford, United Kingdom Mahidol-Oxford Tropical Medicine Research Unit Faculty of Tropical Medicine Mahidol University Bangkok, Thailand; Group A Streptococcal Research Group Murdoch Childrens Research Institute Melbourne, Australia; Group A Streptococcal Research Group Murdoch Childrens Research Institute Centre for International Child Health University of Melbourne Melbourne, Australia

## Abstract

Supplemental Digital Content is available in the text.

## To the Editors:

Group A *Streptococcus* (GAS) is responsible for significant morbidity and mortality globally. Two strong vaccine candidates are currently under evaluation: a 30-valent type–specific M protein–based vaccine and a vaccine targeting the conserved J8 region of M protein.^1^ The 30-valent vaccine covers the most frequent serotypes circulating in high-income countries, but coverage may be suboptimal in low-income settings with greater GAS *emm*-type diversity.^2^ Sixty-eight allelic variants have been described for J8, and the relation between allelic diversity and vaccine efficacy is unclear.^1^ Limited epidemiologic data are available from many regions making vaccine coverage estimates imprecise. There are no previous data for low-income countries in South East Asia.^2^

The clinical microbiology database at Angkor Hospital for Children, a nongovernmental pediatric hospital serving the population of northern Cambodia, was searched to identify clinical GAS isolates cultured between January 1, 2007 and December 31, 2012. These isolates underwent molecular *emm*-typing. *emm*-clusters, and the J8 vaccine antigen content, were deduced from the *emm*-typing result as previously described.^3^ The 30-valent vaccine coverage was estimated using currently available cross-opsonization data.^3,4^ Strain diversity was assessed by Simpson Reciprocal Index.

One hundred fifty GAS isolates from 149 patients were characterized. The median patient age was 3.8 years (range: 0–18.6). One hundred eighteen (78.6%) isolates were from skin and soft tissue infections, 16 (10.7%) from bloodstream infections, 7 (4.7%) from bone/joint infections, 7 (4.7%) from pharyngitis and 2 (1.3%) from infections at other sites. Fifty *emm*-types were identified from 13 *emm*-clusters and 2 isolates were considered nontypeable (see Table, Supplemental Digital Content, http://links.lww.com/INF/C234). No novel *emm*-types were identified. The Simpson Reciprocal Index was 28.5 (95% confidence interval: 23.1–37.3) indicating considerable diversity, similar to that seen in other low-income countries.^2^

Potential coverage of the J8 vaccine was predicted to be excellent with 43 (28.7%) and 104 (69.3%) isolates predicted to have the J8 and J8.1 allele, respectively. Therefore, J8 vaccine coverage could be expected to be 98.0% (95% confidence interval: 94.2%–99.6%). Fifty isolates (33.3%) were of *emm*-types covered by the 30-valent vaccine, and an additional 42 isolates (28.0%) have been shown to be potentially covered by the vaccine as a result of cross-opsonization. Therefore, the potential coverage could be expected to be 61.3% (95% confidence interval: 53.0%–69.2%) but may be higher because 26.0% of the isolates belong to 16 *emm*-types, which have not yet been examined for evidence of cross-opsonization.

Comparison with the only other available regional data set revealed considerably greater *emm*-type diversity in the Cambodian isolates compared with those isolated in Thailand between 1985 and 2004.^5^ Fifty-nine *emm*-types from 13 *emm*-clusters were represented in the combined data set. Only 8 of the 13 *emm*-clusters were found in Thailand. There were 10 shared *emm*-types, comprising 64.2% of the Thailand isolates but only 26.4% of the Cambodia isolates.

Our study had several limitations. Pharyngeal isolates were not well represented because of clinician sampling practices and also there was an absence of adult sampling in the data set. Also, the population studied may not be representative of Cambodia as a whole. Finally, comparisons between the current data and the Thailand data set are limited by the differences between the study populations and nonoverlapping study periods.

Overall, these data indicate a high diversity of circulating GAS strains in Cambodia, the potential high coverage of the J8 vaccine candidate and the need for complementary studies to assess the potential coverage of the 30-valent vaccine candidate. These results highlight the need for robust regional and country-level data for vaccine planning purposes.

## Supplementary Material

**Paul Turner, MB BS, PhD, FRCPCH, FRCPath** Cambodia-Oxford Medical Research Unit Angkor Hospital for Children, Siem Reap, Cambodia, Centre for Tropical Medicine and Global Health Nuffield Department of Medicine University of Oxford Oxford, United Kingdom**Pises Ngeth, MD** Angkor Hospital for Children Siem Reap, Cambodia**Claudia Turner, MB BS, PhD, FRCPCH** Cambodia-Oxford Medical Research Unit Angkor Hospital for Children, Siem Reap, Cambodia Centre for Tropical Medicine and Global Health Nuffield Department of Medicine University of Oxford Oxford, United Kingdom**Sena Sao** Angkor Hospital for Children Siem Reap, Cambodia**Nicholas P. J. Day, MD, FRCP** Centre for Tropical Medicine and Global Health Nuffield Department of Medicine University of Oxford Oxford, United Kingdom Mahidol-Oxford Tropical Medicine Research Unit Faculty of Tropical Medicine Mahidol University Bangkok, Thailand**Ciara Baker, MSc** Group A Streptococcal Research Group Murdoch Childrens Research Institute Melbourne, Australia**Andrew C. Steer, MB BS, PhD, FRACP** **Pierre R. Smeesters, MD, PhD** Group A Streptococcal Research Group Murdoch Childrens Research Institute Centre for International Child Health University of Melbourne Melbourne, Australia
