# Supplementary material for: Molecular Epidemiology of Group A Streptococcus Infections in Cambodian Children, 2007–2012
Source: Pediatr Infect Dis J. 2015 Nov 12;34(12):1414–5. doi: 10.1097/INF.0000000000000878 (PMC4643749; doi:10.1097/INF.0000000000000878)
Supplement: Supplementary file 1 [file inf-34-1414-s001.pdf]

Supplemental Digital Content: Molecular Epidemiology of Group A Streptococcus Infections in Cambodian Children, 2007 - 2012

Paul Turner, Pises Ngeth, Claudia Turner, Sena Sao, Nicholas P.J. Day, Ciara Baker, Andrew C. Steer, Pierre R. Smeesters

emm-clusters and -types of the GAS isolates characterized in the study

***emm*-cluster**	**Isolates (n)**	***emm*-types (n)**
E3	31	9 (4), 25 (4), 44 (14), 58 (1), 58.8 (1), 82.1 (1), 87 (2), 103 (2), 113 (2)
D4	29	33 (1), 41.2 (1), 53 (3), 56 (2), 70 (2), 86.2 (6), 93 (1), 116.1 (1), 119.2 (8), 230 (4)
E6	25	11 (3), 42 (4), 63 (4), 63.3 (1), 67 (2), 75.3 (4), 81.1 (5), 81.2 (1), 85 (1)
E4	21	22 (2), 77 (4), 88.5 (1), 89.14 (6), 102.2 (3), 109.1 (3), 124 (1) 175.1 (1)
A-C3	12	238.1 (11), 239 (1)
E2	11	68 (1), 76 (5), 92 (2), 106 (1), 110 (2)
E1	5	4.2 (1), 4.5 (1), 60.4 (1), 78.3 (2)
M55	5	55 (5)
D2	3	71 (2), 115 (1)
M74	3	74 (3)
A-C4	1	39.4 (1)
D5	1	97.1 (1)
M105	1	105 (1)
Non-typeable	2	Not able to be *emm*-typed (2)
